# Carbachol and Nicotine in Prefrontal Cortex Have Differential Effects on Sleep-Wake States

**DOI:** 10.3389/fnins.2020.567849

**Published:** 2020-11-20

**Authors:** Anjum Parkar, Donald C. Fedrigon, Farah Alam, Giancarlo Vanini, George A. Mashour, Dinesh Pal

**Affiliations:** ^1^Department of Anesthesiology, University of Michigan, Ann Arbor, MI, United States; ^2^Neuroscience Graduate Program, University of Michigan, Ann Arbor, MI, United States; ^3^Center for Consciousness Science, University of Michigan, Ann Arbor, MI, United States

**Keywords:** acetylcholine, carbachol, nicotine, rapid eye movement sleep, slow wave sleep, wakefulness

## Abstract

The role of the brainstem cholinergic system in the regulation of sleep-wake states has been studied extensively but relatively little is known about the role of cholinergic mechanisms in prefrontal cortex in the regulation of sleep-wake states. In a recent study, we showed that prefrontal cholinergic stimulation in anesthetized rat can reverse the traits associated with anesthesia and restore a wake-like state, thereby providing evidence for a causal role for prefrontal cholinergic mechanisms in modulating level of arousal. However, the effect of increase in prefrontal cholinergic tone on spontaneous sleep-wake states has yet to be demonstrated. Therefore, in this study, we tested the hypothesis that delivery of cholinergic agonists – carbachol or nicotine – into prefrontal cortex of rat during slow wave sleep (SWS) would produce behavioral arousal and increase the time spent in wake state. We show that unilateral microinjection (200 nL) of carbachol (1 mM) or nicotine (100 mM) into prefrontal cortex during SWS decreased the latency to the onset of wake state (*p* = 0.03 for carbachol, *p* = 0.03 for nicotine) and increased the latency to the onset of rapid eye movement sleep (*p* = 0.008 for carbachol, *p* = 0.006 for nicotine). Although the infusion of 1 mM carbachol increased the time spent in wake state (*p* = 0.01) and decreased the time spent in SWS (*p* = 0.01), infusion of 10 or 100 mM nicotine did not produce any statistically significant change in sleep-wake architecture. These data demonstrate a differential role of prefrontal cholinergic receptors in modulating spontaneous sleep-wake states.

## Introduction

Studies over the past century have demonstrated that increase in cortical acetylcholine (ACh) is accompanied by electroencephalographic activation and behavioral arousal while decrease in cortical ACh correlates with slow wave sleep (SWS) and anesthetic-induced unconsciousness (Celesia and Jasper, 1966; Phillis, 1968; Jasper and Tessier, 1971; Marrosu et al., 1995; Kikuchi et al., 1998; Shichino et al., 1998; Lydic and Baghdoyan, 2005; Pal et al., 2016). There is also ample evidence from studies conducted across species and laboratories that manipulation of cholinergic tone through systemic or intracranial administration of cholinergic agents (nicotinic or muscarinic) produce changes in electroencephalographic and behavioral arousal (George et al., 1964; Domino and Yamamoto, 1965; Yamamoto and Domino, 1965; Sitaram et al., 1976; Vanni-Mercier et al., 1989; Velazquez-Moctezuma et al., 1990; Baghdoyan et al., 1993; Mallick et al., 2001; Torterolo et al., 2001; Lydic and Baghdoyan, 2005; Alkire et al., 2007; Van Dort et al., 2009; Vanini et al., 2011).

The arousal promoting effect of ACh could be mediated through prefrontal cortex, which has been shown in neuroimaging studies to be highly active during wake state and deactivated during SWS (Maquet et al., 1996; Braun et al., 1997; Nofzinger et al., 1997; Muzur et al., 2002). In a recent study from our laboratory, we demonstrated that reverse dialysis delivery of carbachol, a mixed cholinergic agonist, into rat prefrontal cortex reversed the traits of anesthesia and restored a wake-like state despite the presence of clinically relevant levels of sevoflurane anesthesia (Pal et al., 2018). The carbachol-induced wake-like state was also accompanied by an increase in prefrontal ACh levels (Pal et al., 2018). Although these studies provide compelling evidence in support of a role for prefrontal cortex in arousal, and for cholinergic modulation of behavioral arousal and electroencephalographic activation, evidence for a direct role of prefrontal cholinergic mechanisms in modulating spontaneous sleep-wake states is lacking. Therefore, in this study, we tested the hypothesis that infusion of cholinergic agonists – carbachol and nicotine – into prefrontal cortex of rats during SWS will (i) produce behavioral arousal, (ii) increase wakefulness, and (iii) suppress sleep states. We performed unilateral microinjections of carbachol (1, 10 mM) and nicotine (10, 100 mM), into prefrontal cortex of male Sprague Dawley rats during SWS and measured the effect on (i) latency to the onset of wake state and rapid eye movement (REM) sleep, and (ii) time spent in sleep-wake states. We report that the infusion of carbachol (1 mM) or nicotine (100 mM) into prefrontal cortex decreased the latency to onset of wake state and increased the latency to onset of REM sleep. Infusion of 1 mM carbachol into prefrontal cortex increased wakefulness and decreased SWS whereas infusion of 10 or 100 mM nicotine did not produce any statistically significant effect on sleep-wake states.

## Methods

The experiments were approved by the Institutional Animal Care and Use Committee at the University of Michigan, Ann Arbor and were conducted in compliance with the Guide for the Care and Use of Laboratory Animals (Ed 8, National Academies Press). Adult male Sprague Dawley rats (*n* = 25, 300–350 g, Charles River Inc.) maintained on 12:12 light: dark cycle (lights on at 6:00 am) and with *ad libitum* food and water were used for all the experiments.

### Surgical Procedures

The rats were anesthetized using isoflurane (3–5%) and positioned in a stereotaxic frame (Kopf, David Kopf Inc.) using blunt ear bars. After exposing the cranial surface, holes were drilled for securing bilateral stainless steel screw electrodes for recording electroencephalogram (EEG) from frontal (anterior 3.0 mm, mediolateral 2.5 mm), parietal (posterior 4.0 mm, mediolateral 2.5 mm), and occipital (posterior 8.0 mm, mediolateral 2.5 mm) areas; all coordinates with reference to Bregma. A pair of insulated (except at the tips) wires (AS 636, Cooner Wire Inc.) were positioned into dorsal nuchal muscles to record electromyogram (EMG). In addition, a unilateral stainless steel guide cannula (24G, P1 Technologies) was implanted aimed at the prefrontal cortex (from Bregma: anterior 3.0 mm, mediolateral 0.5 mm, ventral 3.0 mm) (Paxinos and Watson, 2007) for delivery of either carbachol (carbamylcholine chloride, C4382, Millipore-Sigma) or nicotine (nicotine hydrogen tartrate salt, N5260, Millipore-Sigma), and sterile normal saline (918620, Fresenius Kabi) as the vehicle control. The free end of the EEG and EMG electrodes were attached to gold-pins (363A, P1 Technologies), which were routed into two six-pin connectors (MS363, P1 Technologies) and the entire assembly was affixed to the cranial surface using dental cement (51459, Stoelting Dental Cement). Cefazolin (25 mg/kg, subcutaneous) was administered as a pre-surgical antibiotic. Subcutaneous buprenorphine was administered for pre- (0.01 mg/kg) and post- (0.03 mg/kg, every 8–12 h for 48 h) surgical analgesia. The rats were provided 7–10 days for post-surgical recovery and acclimatization to the experimental set-up, during which time the rats were routinely tethered to the EEG/EMG recording cable and habituated to the recording set-up.

### Experimental Design

On the day of experiment, the rats were connected to the EEG/EMG recording cable between 9:30 am – 10:00 am and a stainless steel microinjector (30G, P1 Technologies) was lowered through the implanted guide cannula into prefrontal cortex. The microinjector extended 1.0 mm beyond the implanted guide tube and was connected through polyethylene tubing to a gas-tight syringe (10 μL, Hamilton Inc.) mounted on an automated syringe pump (WPI Inc.). The microinjections (200 nL @ 100 nL per minute) were done remotely around noon after 1 h of pre-injection recording and during SWS without disturbing the animals. SWS was identified by the presence of high-amplitude slow wave EEG and low muscle tone in EMG. The microinjectors, affixed to the recording cables via injection tubing, were left in place till the completion of recording session. The rats were divided into four cohorts: Group 1 received sterile normal saline (vehicle control) and 1 mM Carbachol (*n* = 8 rats), Group 2 received sterile normal saline (vehicle control) and 10 mM Carbachol (*n* = 4 rats), Group 3 received sterile normal saline (vehicle control) and 10 mM Nicotine (*n* = 6 rats), and Group 4 received sterile normal saline (vehicle control) and 100 mM Nicotine (*n* = 7 rats). Each rat received only one injection of saline and one agonist at only one concentration in a counter-balanced order. There was an interval of at least 3–7 days between saline and agonist injections during which the rats were returned to the vivarium but were acclimatized again to the recording set-up at least for a day before the experimental session. The number of rats used in each group was based on our previous studies (Pal and Mallick, 2006, 2009; Pal et al., 2015, 2016). The concentrations for the carbachol and nicotine were based on previously published reports from our (Pal et al., 2018) and other (Crawley et al., 1986; Velazquez-Moctezuma et al., 1990; Nelson et al., 2005) laboratories. The rats in Group 2 showed intense seizures almost immediately after 10 mM carbachol infusion, because of which these experiments were discontinued. The EEG and EMG data were recorded for 1-h pre-injection baseline recording and four post-injection hours.

### Electrophysiological Recording

A Grass model 15 LT bipolar portable physiodata amplifier system (15A54 Quad Amplifier, Natus Neurology) paired with a MP 150 data acquisition unit (Acqknowledge 4.1.1, Biopac Systems Inc.) was used for electroencephalographic and electromyographic recordings. The EEG signals (frontal-frontal, parietal-parietal, and frontal-parietal) were amplified 5000 times, filtered between 0.1 and 300 Hz, and sampled at 1 kHz. The EMG signal was amplified 5000 times, filtered between 0.1 and 100 Hz, and sampled at 250 Hz.

### Sleep-Wake State Identification and Data Analysis

The EEG and EMG data were manually scored using SleepSign (Kissei Comtec Inc.) in 10-s epochs into (1) wake state: low-amplitude fast EEG along with high muscle tone, (2) SWS: high-amplitude slow EEG along with low muscle tone, and (3) REM sleep: low-amplitude fast EEG along with muscle atonia. The percentage of time spent in each state, the mean duration per episode for each state, and the number of episodes per state, after saline and cholinergic agonist injection, were calculated for 1-h pre-injection period and in 1-h bins for 4 post-injection hours. Latency to onset of wakefulness and REM sleep was quantified as the occurrence of the first wake and REM sleep episode, respectively, after the completion of agonist infusion. Supplementary Figure S1A shows representative EEG traces before, during, and after carbachol (1 mM) and nicotine (100 mM) microinjections. The representative sleep-wake states are shown in Supplementary Figure S1B. Supplementary Figure S2 shows the representative EEG and the associated hypnograms for carbachol and nicotine, and the respective saline control injection, for the first post-injection hour.

### Histological Verification of the Site of Microinjection

**FIGURE 1:**
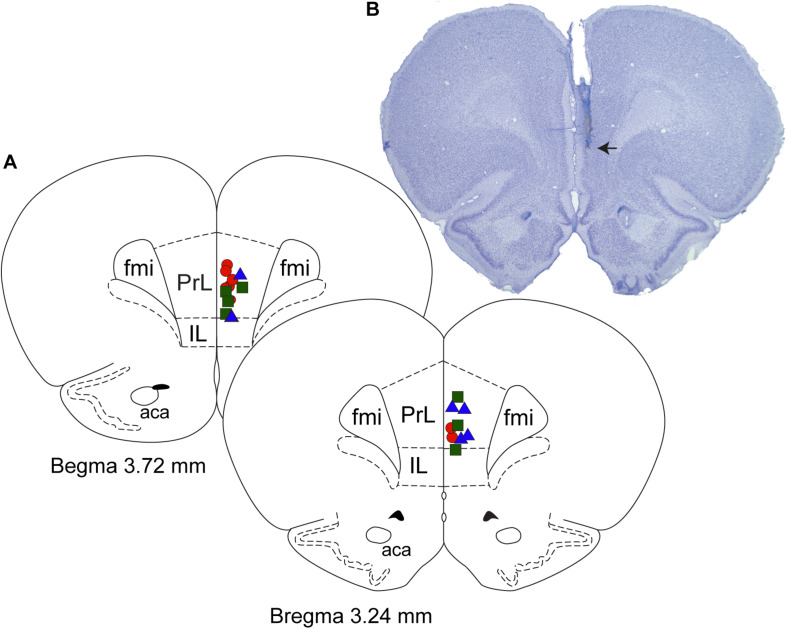
Histological verification of the site of microinjections. **(A)** Coronal brain section drawings show the location of microinjection sites within the prefrontal cortex. Red circles (*n* = 8) – 1 mM carbachol; blue triangles (*n* = 6) – 10 mM nicotine; and green squares (*n* = 7) – 100 mM nicotine. **(B)** Cresyl violet stained representative coronal brain section (30 μm thick) through prefrontal cortex. Arrow shows the site of microinjection. Fmi, forceps minor corpus callosum; IL, infralimbic area; PrL, prelimbic area; aca, anterior commissure.

After the completion of the sleep-wake recording sessions, the rats were euthanized using carbon dioxide and perfused through transcardiac route first with 150 mL of heparinized (1000 units/mL, Heparin: NDC 25021-400-30, Sagent) 0.1 M phosphate-buffered saline (1219SK, Electron Microscopy Sciences) and then with 200 mL of 4% paraformaldehyde in phosphate buffer (15710-S, Electron Microscopy Sciences). The brains were extracted, fixed for 48 h in 4% paraformaldehyde, and then cryoprotected in 30% sucrose (S7903, Millipore-Sigma) for 48–72 h. Each brain was cryosectioned into 30 μm coronal sections through prefrontal cortex using a Leica cryostat (CM1950, Leica) and subsequently stained with 0.5% solution of cresyl violet (AC22963, Fisher Scientific) to visualize the site of microinjection. The histological sections were compared with the photographic plates and stereotaxic diagrams in the rat atlas by Paxinos and Watson (2007) and the sites of injection were plotted as a reconstruction diagram (Figure 1).

### Statistical Analysis

Statistical analyses were conducted in consultation with the Consulting for Statistics, Computing and Analytics Research unit at the University of Michigan (Ann Arbor, Michigan). All statistical comparisons were done using GraphPad Prism (version 8) and the programming and statistical language R (version 4.0.2). A two-tailed paired *t*-test was used for the comparison of latency to onset of wake and REM sleep between the agonist injection and saline sessions. A linear mixed model was used for the comparison of the effect of agonist (1 mM carbachol, 10 mM nicotine, and 100 mM nicotine) injection on the (i) percent time spent in sleep-wake states, (ii) mean duration of each state per episode per hour, and (iii) number of episodes for each state per hour, for each recording hour with the respective saline groups. The linear mixed model was designed with subjects as a random intercept and the agonists, time, and interaction between time and agonist, as the fixed effects. The model was fit with restricted maximum likelihood and accounted for the temporal correlations between the observed changes in sleep-wake states as a fixed effect. A Bonferroni correction was applied to all *post hoc* pairwise comparisons. Each rat received only one saline and one agonist at one concentration. Only the data from Groups 1, 3, and 4 were analyzed. Group 2 was excluded from any analysis because of the carbachol-induced seizures (occurring at 10 mM). The data are reported as mean ± standard error of the mean (SEM) along with 95% confidence interval for the mean.

## Results

The injection sites for the carbachol (1 mM) and nicotine (10 and 100 mM) cohorts were localized to prefrontal cortex (Figure 1). The agonist injection neither had any apparent effect on the raw EEG itself, nor did it produce any dissociated states (Supplementary Figures S1 and S2).

### Carbachol and Nicotine Microinjection Into Prefrontal Cortex Decreased the Latency to the Onset of Wake State and Increased the Latency to the Onset of REM Sleep

**FIGURE 2:**
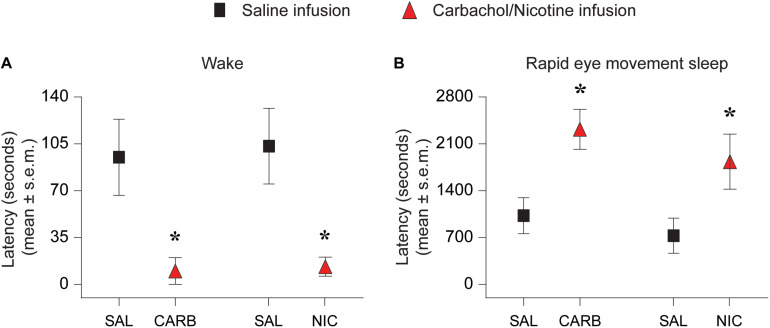
Effect of carbachol (*n* = 6 rats) and nicotine (*n* = 6 rats) delivery into prefrontal cortex on latency to onset of wake state and rapid eye movement sleep. Infusion of 1 mM carbachol (CARB) and 100 mM nicotine (NIC) into prefrontal cortex during slow wave sleep decreased the latency to onset of wake state **(A)** and increased the latency to onset of rapid eye movement sleep **(B)**. The significance symbols denote *p* < 0.05. The actual *p*-values are provided in the text in the results section. *Significant as compared to saline injection. SAL, saline injection; s.e.m., standard error of the mean.

Both carbachol and nicotine showed significant effects on latency to the onset of wake state (Figure 2). In the carbachol group, two rats woke up during saline injection and were excluded from latency analysis. Among the remaining six rats, all but one rat transitioned to wake state immediately after carbachol injection. In the nicotine group, one rat woke up during saline injection, which was excluded from the analysis. From the remaining six rats, three rats transitioned to wake states immediately after the nicotine infusion. Statistical comparison showed that as compared to the saline infusion, carbachol into prefrontal cortex significantly decreased the latency to the onset of wake state [mean ± SEM (95% CI): 95.0 ± 28.5 s (21.8–168.2) for saline vs. 10.0 ± 10.0 s (−15.7 – 35.7) for carbachol, *p* = 0.03, *t*(5) = 3.1] (Figure 2A). Similarly, statistical comparison showed that, compared to the saline infusion, nicotine (100 mM) into prefrontal cortex also significantly decreased the latency to the onset of wake state [mean ± SEM (95% CI): 103.3 ± 28.3 s (30.7 – 175.9) for saline vs. 13.3 ± 7.2 s (−5.0 – 31.7) for nicotine, *p* = 0.03, *t*(5) = 3.1] (Figure 2A). In addition, carbachol significantly increased the latency to onset of REM sleep [mean ± SEM (95% CI): 1027.5 ± 269.2 s (390.9–1664.1) for saline vs. 2317.5 ± 299.2 s (1610.1–3024.9) for carbachol, *p* = 0.008, *t*(7) = 3.7] (Figure 2B). Similar to carbachol, nicotine also produced a significant increase in latency to onset of REM sleep [mean ± SEM (95% CI): 728.6 ± 262.1 s (87.1–1370.0) for saline vs. 1834.3 ± 411.3 s (827.9–2840.6) for nicotine, *p* = 0.006, *t*(6) = 4.2] (Figure 2B). There was no statistical effect of 10 mM nicotine on latency to onset of wake state or REM sleep.

### Carbachol Microinjection Into Prefrontal Cortex Increased Wakefulness and Decreased SWS

**FIGURE 3:**
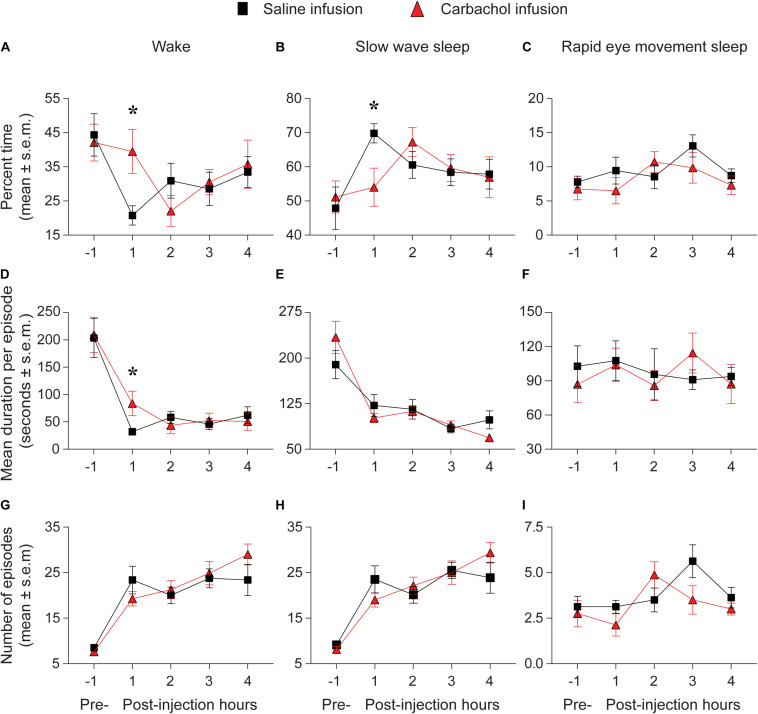
Carbachol microinjection (*n* = 8 rats) into prefrontal cortex increased wakefulness and decreased slow wave sleep. Percent time spent in **(A)** wake state, **(B)** slow wave sleep, and **(C)** rapid eye movement sleep during pre-injection 1 hour and after saline (black squares) or 1 mM carbachol (red triangles) injection into prefrontal cortex. The panels **(D–F)** show the mean duration per episode during pre-injection 1 h and for each of the four post-injection hours for wake **(D)**, slow wave sleep **(E)**, and rapid eye movement sleep **(F)**. The number of episodes during pre-injection 1 h and for each of the four post-injection hours are shown in **(G)** (wake), **(H)** (slow wave sleep), and **(I)** (rapid eye movement sleep). The significance symbols denote *p* < 0.05. The actual *p*-values are provided in the text in the results section. *Significant as compared to saline injection. Pre-, pre-injection 1-h period; s.e.m., standard error of the mean.

The percent time spent in sleep-wake states during the pre-injection 1-h period was not significantly different between the saline and 1 mM carbachol injection group (Figure 3). As compared to saline infusion, carbachol (1 mM) delivery into prefrontal cortex produced a significant increase in time spent in wake state during the first post-injection hour [mean ± SEM (95% CI): 21.7 ± 5.4% (10.9–32.4) for saline vs. 40.2 ± 5.3% (29.7–50.8) for carbachol, *p* = 0.01] (Figure 3A). There was no statistically significant difference in the percent time spent in wake state between the saline and carbachol infusion groups for the remaining three post-injection hours [mean ± SEM (95% CI): hour 2 = 30.0 ± 5.3% (19.3–40.7) for saline vs. 22.6 ± 5.2% (12.2–33.0) for carbachol, *p* = 0.34; hour 3 = 28.4 ± 5.1% (18.3–38.6) for saline vs. 29.7 ± 5.3% (19.2–40.3) for carbachol, *p* = 0.86; hour 4 = 33.2 ± 5.1% (22.9–43.4) for saline vs. 35.6 ± 5.1% (25.4–45.8) for carbachol, *p* = 0.73] (Figure 3A). Further analysis of the changes in architecture of sleep-wake states showed that the carbachol-induced increase in wakefulness during first post-injection hour was due to a significant increase in the mean duration per episode [mean ± SEM (95% CI): 36.6 ± 17.9 s (0.7–72.4) for saline vs. 88.5 ± 18.2 s (51.9–125.1) for carbachol, *p* = 0.01] (Figure 3D). The saline and carbachol infusion groups did not show any statistically significant difference in the mean duration per episode for the remaining three post-injection hours [mean ± SEM (95% CI): hour 2 = 55.9 ± 15.4 s (25.0–86.7) for saline vs. 43.3 ± 14.3 s (14.7–72.0) for carbachol, *p* = 0.55; hour 3 = 44.5 ± 14.6 s (15.2–73.9) for saline vs. 50.6 ± 15.0 s (20.5–80.6) for carbachol, *p* = 0.77; hour 4 = 60.4 ± 14.9 s (30.5–90.3) for saline vs. 48.9 ± 14.8 s (19.4–78.5) for carbachol, *p* = 0.57] (Figure 3D) or in the number of wake episodes for any of the four post-injection hours [mean ± SEM (95% CI): hour 1 = 26.0 ± 2.9 (20.2–31.7) for saline vs. 22.1 ± 2.9 (16.1–28.0) for carbachol, *p* = 0.21; hour 2 = 18.8 ± 2.5 (13.8–23.7) for saline vs. 21.1 ± 2.3 (16.4–25.7) for carbachol, *p* = 0.47; hour 3 = 23.4 ± 2.3 (18.7–28.1) for saline vs. 24.2 ± 2.4 (19.4–28.9) for carbachol, *p* = 0.79; hour 4 = 22.0 ± 2.5 (17.1–27.0) for saline vs. 27.4 ± 2.6 (22.3–32.5) for carbachol, *p* = 0.09] (Figure 3G). During the first post-injection hour, we also observed statistically significant decrease in the time spent in SWS [mean ± SEM (95% CI): 69.9 ± 4.7% (60.4–79.3) for saline vs. 54.1 ± 4.6% (44.9–63.3) for carbachol, *p* = 0.01] (Figure 3B). There was no statistically significant difference in the percent time spent in SWS between the saline and carbachol infusion groups for the remaining three post-injection hours [mean ± SEM (95% CI): hour 2 = 60.5 ± 4.8% (51.0–70.0) for saline vs. 67.3 ± 4.5% (58.2–76.4) for carbachol, *p* = 0.29; hour 3 = 58.4 ± 4.5% (49.4–67.4) for saline vs. 59.5 ± 4.6% (50.2–68.9) for carbachol, *p* = 0.85; hour 4 = 57.8 ± 4.5% (48.8–66.8) for saline vs. 56.9 ± 4.5% (47.9–65.9) for carbachol, *p* = 0.88] (Figure 3B). There was no statistically significant difference between the saline and carbachol group, in any of the four post-injection hours, for the mean duration of SWS per episode [mean ± SEM (95% CI): hour 1 = 117.7 ± 12.8 s (92.1–143.4) for saline vs. 94.0 ± 15.2 s (63.4–124.5) for carbachol, *p* = 0.13; hour 2 = 116.4 ± 11.5 s (93.3–139.6) for saline vs. 114.4 ± 11.8 s (90.6–138.2) for carbachol, *p* = 0.89; hour 3 = 85.2 ± 11.6 s (62.0–108.5) for saline vs. 91.1 ± 11.6 s (67.8–114.5) for carbachol, *p* = 0.69; hour 4 = 101.7 ± 12.3 s (76.9–126.5) for saline vs. 71.9 ± 12.1 s (47.5–96.3) for carbachol, *p* = 0.06] (Figure 3E) or the number of SWS episodes [mean ± SEM (95% CI): hour 1 = 25.4 ± 2.9 (19.7–31.2) for saline vs. 21.1 ± 2.9 (15.1–27.1) for carbachol, *p* = 0.17; hour 2 = 19.3 ± 2.4 (14.4–24.1) for saline vs. 22.1 ± 2.3 (17.5–26.8) for carbachol, *p* = 0.37; hour 3 = 25.3 ± 2.3 (20.7–29.9) for saline vs. 24.4 ± 2.4 (19.7–29.2) for carbachol, *p* = 0.77; hour 4 = 22.6 ± 2.6 (17.5–27.8) for saline vs. 28.2 ± 2.5 (23.2–33.3) for carbachol, *p* = 0.07] (Figure 3H). Compared to the saline infusion group, infusion of carbachol into prefrontal cortex did not produce any statistically significant change in the time spent in REM sleep in any of the four post-injection hours [mean ± SEM (95% CI): hour 1 = 9.5 ± 1.7% (6.1–13.0) for saline vs. 6.6 ± 1.8% (3.1–10.1) for carbachol, *p* = 0.23; hour 2 = 8.5 ± 1.7% (5.1–12.0) for saline vs. 10.9 ± 1.8% (7.3–14.4) for carbachol, *p* = 0.34; hour 3 = 13.1 ± 1.7% (9.7–16.5) for saline vs. 9.77 ± 1.7% (6.3–13.2) for carbachol, *p* = 0.17; hour 4 = 8.5 ± 1.8% (4.9–12.1) for saline vs. 7.3 ± 1.7% (3.8–10.7) for carbachol, *p* = 0.62] (Figure 3C). There was no significant effect of carbachol, in any of the four post-injection hours, on either the mean duration per episode for REM sleep [mean ± SEM (95% CI): hour 1 = 107.4 ± 15.6 s (76.0–139.0) for saline vs. 105.0 ± 15.7 s (73.5–137.0) for carbachol, *p* = 0.92; hour 2 = 94.9 ± 15.7 s (63.5–126.0) for saline vs. 85.4 ± 15.7 s (54.0–117.0) for carbachol, *p* = 0.67; hour 3 = 91.3 ± 15.6 s (59.9–123.0) for saline vs. 115.6 ± 15.7 s (84.1–147.0) for carbachol, *p* = 0.28; hour 4 = 94.5 ± 15.7 s (63.1–126.0) for saline vs. 85.8 ± 15.8 s (54.2–117.0) for carbachol, *p* = 0.69] (Figure 3F) or the number of REM sleep episodes [mean ± SEM (95% CI): hour 1 = 3.1 ± 0.7 (1.8–4.4) for saline vs. 2.1 ± 0.7 (0.8–3.4) for carbachol, *p* = 0.26; hour 2 = 3.5 ± 0.7 (2.2–4.8) for saline vs. 4.8 ± 0.7 (3.4–6.1) for carbachol, *p* = 0.17; hour 3 = 5.6 ± 0.6 (4.3–6.9) for saline vs. 3.6 ± 0.7 (2.3–4.9) for carbachol, *p* = 0.06; hour 4 = 3.8 ± 0.7 (2.4–5.2) for saline vs. 2.9 ± 0.7 (1.7–4.3) for carbachol, *p* = 0.40] (Figure 3I).

Unlike the effect of 1 mM carbachol on altering sleep-wake states, infusion of 10 mM carbachol into prefrontal cortex during SWS caused immediate arousal and behavioral seizures. We found the same reproducible effect in four rats and therefore stopped the studies using 10 mM carbachol.

### Nicotine Microinjection Into Prefrontal Cortex Had no Significant Effect on Sleep-Wake States

The percent time spent in sleep-wake states during the pre-injection 1-h period was not significantly different between the saline and nicotine injection group (Table 1). Infusion of 100 mM nicotine produced no significant change in the time spent in any of the sleep-wake states (Table 1). Furthermore, there was no significant difference in the mean duration per episode or the number of episodes for any of the sleep-wake states (Table 1). As compared to the saline infusion, 10 mM nicotine into prefrontal cortex also did not produce any statistically significant change in the time spent in wake or SWS (Supplementary Table S1). There was no significant change in the mean duration per episode and the number of episodes for the wake state and SWS. The time spent in REM sleep followed the same trend as with wake state and SWS except that there was a statistically significant decrease during the third post-injection hour [mean ± SEM (95% CI): 16.5 ± 1.9% (12.6–20.3) for saline vs. 9.7 ± 1.9% (5.9–13.6) for nicotine, *p* = 0.02]. (Supplementary Table S1).

**TABLE 1:** Effect of 100 mM nicotine (NIC) and saline (SAL) delivery into prefrontal cortex (*n* = 7 rats) on sleep-wake states.

Hours		Percent time	*p*-Value	Duration per episode	*p*-Value	Number of episodes	*p*-Value
						
		Mean ± SEM (95% CI)		Mean ± SEM (95% CI)		Mean ± SEM (95% CI)	
**Wake**							
−1	NIC	36.6 ± 8.0 (16.9–56.3)	0.11	87.9 ± 27.9 (19.4–156.3)	0.06	17.1 ± 2.8 (10.3–24.0)	0.15
	SAL	50.3 ± 4.7 (38.8–61.8)		143.4 ± 17.5 (100.5–186.4)		13.1 ± 1.1 (10.4–15.9)	
1	NIC	27.6 ± 5.4 (16.7–38.6)	0.15	54.6 ± 7.2 (40.0–69.1)	0.08	19.0 ± 2.5 (13.9–24.1)	0.83
	SAL	16.6 ± 6.3 (3.9–29.3)		35.1 ± 9.6 (15.8–54.5)		18.3 ± 2.6 (13.0–23.6)	
2	NIC	22.0 ± 5.3 (11.4–32.6)	0.74	37.3 ± 6.9 (23.4–51.1)	0.87	21.3 ± 2.5 (16.2–26.4)	0.61
	SAL	19.5 ± 5.5 (8.4–30.6)		35.6 ± 7.1 (21.3–49.9)		19.5 ± 2.5 (14.4–24.6)	
3	NIC	25.9 ± 5.3 (15.15–36.6)	0.61	45.2 ± 7.1 (30.8–59.5)	0.73	19.1 ± 2.6 (13.9–24.3)	0.88
	SAL	22.2 ± 5.4 (11.3–33.0)		41.8 ± 7.2 (27.4–56.2)		18.6 ± 2.5 (13.5–23.7)	
4	NIC	38.3 ± 5.3 (27.7–48.9)	0.15	55.3 ± 6.9 (41.2–69.3)	0.62	22.8 ± 2.5 (17.6–27.9)	0.38
	SAL	27.4 ± 5.3 (16.7–38.2)		50.3 ± 7.0 (36.2–64.5)		19.8 ± 2.5 (14.7–24.9)	
**Slow wave sleep**							
−1	NIC	56.1 ± 6.6 (40.1–72.2)	0.12	125.1 ± 23.9 (66.7–183.6)	0.47	18.3 ± 2.8 (11.5–25.0)	0.11
	SAL	44.8 ± 4.1 (34.8–54.7)		122.6 ± 15.4 (85.0–160.1)		13.6 ± 1.1 (10.9–16.2)	
1	NIC	67.2 ± 4.4 (58.3–76.1)	0.37	128.2 ± 16.3 (95.0–161)	0.48	21.2 ± 2.4 (16.5–26.0)	0.46
	SAL	73.0 ± 5.1 (62.8–83.2)		142.4 ± 16.3 (109.2–176)		23.7 ± 2.6 (18.5–28.8)	
2	NIC	66.5 ± 4.6 (57.3–75.8)	0.59	110.2 ± 16.4 (76.9–143)	0.99	24.0 ± 2.3 (19.3–28.7)	0.89
	SAL	63.3 ± 4.4 (54.4–72.1)		110.1 ± 16.6 (76.4–144)		23.6 ± 2.3 (18.9–28.3)	
3	NIC	57.7 ± 4.4 (49.0–66.5)	0.34	95.6 ± 16.3 (62.4–129)	0.50	24.5 ± 2.4 (19.7–29.2)	0.36
	SAL	63.7 ± 4.4 (54.7–72.6)		109.1 ± 16.3 (76.0–142)		21.5 ± 2.4 (16.7–26.2)	
4	NIC	49.7 ± 4.4 (40.9–58.5)	0.30	86.2 ± 16.6 (52.4–120)	0.50	23.2 ± 2.4 (18.3–28.1)	0.64
	SAL	56.2 ± 4.4 (47.5–65.0)		99.7 ± 16.4 (66.4–133)		21.7 ± 2.4 (16.9–26.4)	
**Rapid eye movement sleep**							
−1	NIC	7.3 ± 2.1 (2.1–12.3)	0.07	66.9 ± 17.4 (24.3–109.5)	0.21	3.1 ± 0.9 (0.8–5.5)	0.08
	SAL	4.9 ± 1.3 (1.8–8.2)		82.1 ± 21.8 (28.8–135.5)		1.7 ± 0.5 (0.6–2.9)	
1	NIC	7.9 ± 3.3 (1.2–14.6)	0.11	75.1 ± 15.8 (43.0–107.0)	0.91	3.1 ± 1.1 (0.9–5.4)	0.06
	SAL	15.4 ± 3.4 (8.5–22.2)		77.4 ± 15.7 (45.6–109.0)		6.3 ± 1.2 (3.9–8.7)	
2	NIC	17.3 ± 3.4 (10.5–24.1)	0.37	107.9 ± 15.7 (76.0–140.0)	0.95	5.5 ± 1.1 (3.2–7.8)	0.90
	SAL	12.9 ± 3.3 (6.4–19.5)		109.0 ± 15.7 (77.2–141)		5.7 ± 1.1 (3.5–7.9)	
3	NIC	14.9 ± 3.3 (8.4–21.6)	0.29	74.8 ± 15.9 (42.5–107.0)	0.76	7.8 ± 1.1 (5.7–9.9)	0.55
	SAL	19.9 ± 3.3 (13.4–26.5)		68.8 ± 16.0 (36.4–101.0)		6.9 ± 1.1 (4.7–9.1)	
4	NIC	10.1 ± 3.3 (3.4–16.8)	0.75	73.3 ± 15.7 (41.4–105)	0.06	5.3 ± 1.2 (2.9–7.7)	0.72
	SAL	11.6 ± 3.5 (4.5–18.7)		112.5 ± 15.7 (80.5–144.0)		4.8 ± 1.1 (2.5–7.0)	

*CI, confidence intervals of the mean. SEM, standard error of the mean. Note that injection hour −1 is the pre-injection hour. Hours 1–4 are the post-injection hours.*

## Discussion

The main finding of this study is that the infusion of carbachol into prefrontal cortex produced a short-lasting increase in the time spent in wake state and decrease in SWS. Carbachol also decreased the latency to the onset of wake state and increased the latency to the onset of REM sleep. In contrast to carbachol infusion, nicotine delivery into prefrontal cortex did not produce overall changes in sleep-wake states. However, similar to carbachol, nicotine also decreased the latency to the onset of wake state and increased the latency to the onset of REM sleep. The differential effect of carbachol and nicotine on sleep-wake states confirms that these effects are receptor specific and discounts the possibility of having a non-specific increase in wakefulness due to agonist infusion *per se*. The possible methodological confounds were further minimized by using a remote pump for all microinjections. Previous *in vivo* and *in vitro* studies using co-application of carbachol and atropine (Bourgin et al., 1995; Capece et al., 1998; Marks and Birabil, 1998; Demarco et al., 2004; Nelson et al., 2005) as well as direct iontophoretic co-application of nicotine and carbachol with their antagonists into prefrontal cortex (Vidal and Changeux, 1989) demonstrate the specificity of these agonists at the concentrations used in this study.

Carbachol is a highly potent cholinomimetic agent, but because it does not cross the blood brain barrier it has primarily been administered intracranially to investigate the role of cholinergic mechanisms in sleep-wake states. Delivery of carbachol into the pontine tegmentum (Baghdoyan et al., 1987; Torterolo et al., 2001; Lydic and Baghdoyan, 2005) and locus coeruleus (Mallick et al., 2001) was shown to increase REM sleep whereas infusion of carbachol into basal forebrain decreases SWS and increases wakefulness (Baghdoyan et al., 1993). Demarco et al. (2004) demonstrated that carbachol injection into the pontine reticular formation of anesthetized mouse decreased ACh levels in the prefrontal cortex and increased the time to resumption of righting reflex, both suggestive of a decrease in arousal levels. Increasing ACh levels in the prefrontal cortex of anesthetized mouse was shown to produce EEG activation, decrease EEG delta power, and reduce the time to resumption of righting reflex, all suggestive of increased behavioral arousal (Van Dort et al., 2009). Considering these earlier findings (Kodama et al., 1990; Demarco et al., 2004; Van Dort et al., 2009), our data are consistent with an antagonistic relationship between prefrontal cortex and pontine reticular formation, wherein increase in prefrontal ACh suppresses pontine ACh levels and produces wakefulness, while increase in pontine ACh levels suppress prefrontal ACh release and promotes REM sleep. A similar antagonistic relationship between basal forebrain and pontine reticular formation was posited by Baghdoyan et al. (1993). Of note, there is reciprocal connectivity among prefrontal cortex, cholinergic basal forebrain, and the cholinergic laterodorsal/pedunculopontine tegmentum that innervates pontine reticular formation (Baghdoyan et al., 1993; Briand et al., 2007). This connectivity pattern forms a tripartite circuitry with causal relevance for sleep-wake states.

Systemic and intracranial administration of nicotine and cholinomimetics have also been shown to promote behavioral arousal. Intravenous administration of nicotine during SWS in cats produced immediate electroencephalographic activation and behavioral arousal (Domino and Yamamoto, 1965; Yamamoto and Domino, 1965). Intravenous infusion of physostigmine – an acetylcholinesterase inhibitor with both central and peripheral effects – at the onset of REM sleep in human subjects produced significantly more awakenings and with higher frequency (Sitaram et al., 1976). Bilateral microinjection of nicotine into central medial thalamus (Alkire et al., 2007) and reverse dialysis delivery of carbachol into prefrontal cortex (Pal et al., 2018) of anesthetized rat was shown to reverse the state of anesthesia and produce a wake-like state. The effect of cholinergic agents on state transitions, as previously reported in the above discussed studies, was also found in the current study. We observed that carbachol and nicotine administration in prefrontal cortex during SWS decreased the latency to the onset of wake state (five out of six animals woke up immediately after carbachol infusion and three out of six animals woke up immediately after nicotine infusion) and increased the latency to the onset of REM sleep. Increase in REM sleep latency has also been reported after systemic delivery of nicotine in rats (Salin-Pascual et al., 1999; Vázquez-Palacios et al., 2010), and although the difference in route of administration precludes any direct comparison, it is possible that the increase in latency to the onset of REM sleep as reported earlier with systemic nicotine administration is at least partly mediated through prefrontal cortex.

The prefrontal cortex receives cholinergic innervation from basal forebrain and laterodorsal/pedunculopontine tegmentum (Saper and Loewy, 1982; Vincent et al., 1983; Satoh and Fibiger, 1986; Chaves-Coira et al., 2018), both of which have wake-active and REM sleep-active neurons (Lee et al., 2005; Sakai, 2012; Boucetta et al., 2014). Although the role of prefrontal ACh from either of these sources in triggering the onset of wake state is not clear, recent optogenetic studies showed that the stimulation of basal forebrain cholinergic neurons induced wakefulness (Irmak and de Lecea, 2014; Xu et al., 2015) whereas the stimulation of PPT cholinergic neurons primarily increased REM sleep (Van Dort et al., 2015). Stimulation of basal forebrain cholinergic neurons has also been reported to increase local ACh levels and wakefulness while simultaneous local delivery of atropine was reported to attenuate the wake-promoting effect of cholinergic stimulation, thereby suggesting that basal forebrain cholinergic neurons likely act through local non-cholinergic, including the parvalbumin positive GABAergic, neurons to promote wakefulness (Zant et al., 2016). Of note, ACh in basal forebrain has been shown to excite cortically projecting parvalbumin positive GABAergic neurons (Yang et al., 2014). Furthermore, recent chemogenetic and optogenetic studies have demonstrated an executive role for cortically projecting GABAergic neurons in basal forebrain in promoting wakefulness and arousal (Anaclet et al., 2015; Xu et al., 2015; McKenna et al., 2020). In view of these data, it is possible that ACh in prefrontal cortex excite or disinhibit the local pyramidal neurons, which send glutamatergic projections primarily to non-cholinergic, including GABAergic, neurons in basal forebrain (Zaborszky et al., 1997). Excitation of GABAergic neurons in basal forebrain either directly through prefrontal glutamatergic projections or indirectly through local cholinergic neurons, may induce or enhance wakefulness.

It is important to note that the effect of carbachol in our study was primarily limited to the first post-injection hour. The relatively short-lasting effect could be due, in part, to the use of unilateral microinjections instead of bilateral injections as well as limited spread of the injection volume (200 nL) in prelimbic region, which has an anteroposterior expanse of more than 2 mm and mediolateral expanse of about 1 mm. We could not increase the carbachol concentration beyond 1 mM because at higher concentration (10 mM) it induced behavioral seizures, which corroborates previous reports of behavioral seizures and epileptiform activity after infusion of carbachol into prefrontal cortex (Crawley et al., 1986; Stivers et al., 1988; Pal et al., 2018), and highlights the pharmacological limit for the use of carbachol. Another potential limitation of our study is that we did not investigate the effects of these cholinergic agents in another cortical site on sleep-wake states. However, in a recent study in anesthetized rat (Pal et al., 2018), we demonstrated that dialysis delivery of carbachol into prefrontal cortex, but not two distinct sites in parietal cortex, reversed the state of anesthesia and produced a wake-like state, thus demonstrating the specificity of arousal promoting effects of carbachol to prefrontal cortex. Additionally, the use of cholinergic antagonists could have supplemented these data and further validated the role of prefrontal cholinergic system in sleep-wake states, which is a potential limitation in our study. However, cholinergic agonism and antagonism in the regulation of arousal states cannot be assumed to be mirror images of one another. For example, although nicotine infusion in central medial thalamus promoted a transition from anesthetized to wake state, infusion of mecamylamine – a nicotine antagonist – in the same location had no effect in facilitating transition from waking to the anesthetized state (Alkire et al., 2007). Similarly, pretreatment with atropine prevented the REM sleep-promoting effect of carbachol in the pontine region, but the infusion of atropine by itself into the same site in the same studies did not alter sleep-wake states (Bourgin et al., 1995; Marks and Birabil, 1998).

## Conclusion

In summary, we show that carbachol but not nicotine in prefrontal cortex, promote wakefulness and suppresses sleep. Despite the relatively short-lasting effects, our data demonstrate a role for prefrontal cholinergic receptors in modulating spontaneous sleep-wake states. These causal data prompt further mechanistic studies to dissect the role of muscarinic and nicotinic receptor subtypes in prefrontal cortex in sleep-wake states.

## Data Availability Statement

The raw data supporting the conclusions of this article can be made available on request. Requests to access these datasets should be directed to, DP, dineshp@med.umich.edu.

## Ethics Statement

The animal study was reviewed and approved by the Institutional Animal Care and Use Committee, University of Michigan, Ann Arbor.

## Author Contributions

AP analyzed the data and wrote the manuscript. DF and FA performed the experiments and analyzed the data. GV and GM wrote the manuscript. GM and DP designed the experiments. DP performed the experiments, analyzed the data, and wrote the manuscript. All authors contributed to the article and approved the submitted version.

## Conflict of Interest

The authors declare that the research was conducted in the absence of any commercial or financial relationships that could be construed as a potential conflict of interest.

## Supplementary Material

Supplementary Figure 1 **(A)** Representative electroencephalograms before, during, and after 1 mM carbachol or 100 mM nicotine injection into prefrontal cortex. **(B)** Representative electroencephalograms and electromyogram (EMG) across sleep-wake states. The electroencephalograms are from bipolar montage between two frontal (Frtl) or two parietal (Prtl) recording electrodes.

Supplementary Figure 2 Representative electroencephalograms and the associated hypnograms show the effect of **(A)** 1 mM carbachol, and **(B)** 100 mM nicotine microinjection into prefrontal cortex on sleep-wake states during the first post-injection hour. The electroencephalograms are from bipolar montage between two frontal (Frtl) or two parietal (Prtl) recording electrodes. The horizontal bar on top left of electroencephalograms show the duration of injection (120 s). REMS, rapid eye movement sleep; SWS, slow wave sleep.

Supplementary Table 1 Effect of 10 mM nicotine (NIC) and saline (SAL) delivery into prefrontal cortex (*n* = 6 rats) on sleep-wake states.
