# Supplementary material for: Carbachol and Nicotine in Prefrontal Cortex Have Differential Effects on Sleep-Wake States
Source: Front Neurosci. 2020 Nov 20;14:567849. doi: 10.3389/fnins.2020.567849 (PMC7714754; doi:10.3389/fnins.2020.567849)
Supplement: Supplementary Table 1 — Effect of 10 mM nicotine (NIC) and saline (SAL) delivery into prefrontal cortex (n = 6 rats) on sleep-wake states. [file Table_1.docx]

**Supplementary Table S1.** Effect of 10 mM nicotine (NIC) and saline (SAL) delivery into prefrontal cortex (n=6 rats) on sleep-wake states.

| **Wake** | | | | | | | |
| --- | --- | --- | --- | --- | --- | --- | --- |
| Hours | | Percent time  mean ± s.e.m (95% CI) | p  value | Duration per episode  mean ± s.e.m (95% CI) | p value | Number of episodes  mean ± s.e.m (95% CI) | p value |
| -1 | NIC | 20.2 ± 4.6 (8.5-31.9) | 0.07 | 35.2 ± 3.9 (24.9-45.4) | 0.02* | 20.2 ± 3.7 (10.7-29.6) | 0.26 |
|  | SAL | 30.3 ± 2.8 (23.2-37.4) |  | 83.2 ± 16.5 (40.7-125.6) |  | 15.8 ± 3.5 (6.8-24.9) |  |
| 1 | NIC | 26.6 ± 6.5 (13.3-39.8) | 0.79 | 39.5 ± 14.2 (10.8-68.2) | 0.47 | 22.6 ± 3.2 (15.9-29.4) | 0.92 |
|  | SAL | 28.7 ± 6.3 (15.8-41.6) |  | 55.0 ± 14.9 (25.0-85.1) |  | 22.3 ± 3.3 (15.4-29.2) |  |
| 2 | NIC | 33.3 ± 6.3 (20.3-46.2) | 0.26 | 64.6 ± 14.1 (36.1-93.1) | 0.31 | 22.9 ± 3.2 (16.2-29.6) | 0.34 |
|  | SAL | 24.6 ± 6.3 (11.7-37.5) |  | 44.1 ± 13.9 (16.0-72.2) |  | 19.5 ± 3.2 (12.8-26.2) |  |
| 3 | NIC | 36.8 ± 6.3 (23.9-49.8) | 0.33 | 55.8 ± 14.0 (27.5-84.1) | 0.47 | 23.2 ± 3.2 (16.4-29.9) | 0.38 |
|  | SAL | 29.1 ± 6.3 (16.2-42.1) |  | 41.4 ± 13.9 (13.2-69.6) |  | 26.4 ± 3.2 (19.6-33.1) |  |
| 4 | NIC | 27.7 ± 6.5 (14.3-41.1) | 0.17 | 43.4 ± 13.9 (15.3-71.5) | 0.19 | 25.6 ± 3.2 (18.9-32.4) | 0.17 |
|  | SAL | 38.8 ± 6.3 (25.9-51.6) |  | 70.1 ± 14.0 (41.8-98.5) |  | 20.7 ± 3.3 (13.8-27.5) |  |
| **Slow wave sleep** | | | | | | | |
| Hours | | Percent time  mean ± s.e.m (95% CI) | p  value | Duration per episode  mean ± s.e.m (95% CI) | p value | Number of episodes  mean ± s.e.m (95% CI) | p value |
| -1 | NIC | 71.4 ± 4.3 (60.4-82.3) | 0.17 | 128.5 ± 22.6 (70.3-186.7) | 0.27 | 22.5 ± 3.2 (14.3-30.7) | 0.19 |
|  | SAL | 63.8 ± 3.9 (53.7-73.9) |  | 159.3 ± 31.9 (77.3-241.3) |  | 17.2 ± 3.2 (8.8-25.5) |  |
| 1 | NIC | 61.1 ± 5.7 (49.3-72.9) | 0.81 | 96.6 ± 20.0 (55.2-138.0) | 0.78 | 24.7 ± 3.1 (18.4-31.0) | 0.57 |
|  | SAL | 59.6 ± 5.4 (48.2-70.9) |  | 103.3 ± 20.8 (60.3-146.0) |  | 26.9 ± 3.2 (20.2-33.5) |  |
| 2 | NIC | 55.4 ± 5.5 (44.1-66.7) | 0.19 | 87.7 ± 19.8 (46.6-129.0) | 0.36 | 25.0 ± 3.1 (18.8-31.3) | 0.55 |
|  | SAL | 63.7 ± 5.4 (52.4-75.0) |  | 108.9 ± 19.8 (67.8-150.0) |  | 22.9 ± 3.1 (16.6-29.2) |  |
| 3 | NIC | 54.0 ± 5.5 (42.6-65.4) | 0.94 | 82.1 ± 20.0 (40.6-124.0) | 0.70 | 24.6 ± 3.1 (18.3-31.0) | 0.24 |
|  | SAL | 54.4 ± 5.4 (43.1-65.7) |  | 91.1 ± 19.8 (50.0-132.0) |  | 29.0 ± 3.1 (22.7-35.3) |  |
| 4 | NIC | 59.6 ± 5.6 (48.0-71.2) | 0.13 | 90.0 ± 20.3 (48.1-132.0) | 0.85 | 26.5 ± 3.1 (20.2-32.8) | 0.36 |
|  | SAL | 49.8 ± 5.5 (38.3-61.2) |  | 85.6 ± 19.9 (44.2-127.0) |  | 23.1 ± 3.1 (16.7-29.6) |  |
| **Rapid eye movement sleep** | | | | | | | |
| Hours | | Percent time  mean ± s.e.m (95% CI) | p value | Duration per episode  mean ± s.e.m (95% CI) | p  value | Number of episodes  mean ± s.e.m (95% CI) | p value |
| -1 | NIC | 8.4 ± 1.0 (5.7-11.0) | 0.14 | 76.5 ± 9.6 (51.9-101.1) | 0.37 | 4.0 ± 0.4 (3.1-4.9) | 0.1 |
|  | SAL | 5.9 ± 1.5 (2.1-9.9) |  | 82.5 ± 22.2 (25.5-139.5) |  | 2.7 ± 0.7 (0.8-4.5) |  |
| 1 | NIC | 10.9 ± 1.9 (6.9-14.9) | 0.87 | 74.2 ± 15.1 (43.6-104.7) | 0.88 | 5.9 ± 1.3 (3.3-8.5) | 0.93 |
|  | SAL | 10.5 ± 2.1 (6.2-14.7) |  | 78.0 ± 15.1 (47.5-108.6) |  | 6.0 ± 1.4 (3.3-8.8) |  |
| 2 | NIC | 11.1 ± 1.9 (7.2-14.9) | 0.81 | 82.6 ± 15.1 (52.1-113.2) | 0.68 | 5.7 ± 1.2 (3.2-8.2) | 0.69 |
|  | SAL | 11.7 ± 1.9 (7.9-15.6) |  | 91.5 ± 15.1 (61.0-122.1) |  | 6.4 ± 1.2 (3.9-8.9) |  |
| 3 | NIC | 9.7 ± 1.9 (5.9-13.6) | 0.02* | 54.6 ± 15.1 (24.0-85.1) | 0.19 | 6.3 ± 1.2 (3.8-8.8) | 0.40 |
|  | SAL | 16.5 ± 1.9 (12.6-20.3) |  | 83.0 ± 15.2 (52.2-113.8) |  | 7.8 ± 1.3 (5.3-10.3) |  |
| 4 | NIC | 13.2 ± 1.9 (9.4-17.1) | 0.99 | 67.4 ± 15.5 (36.0-98.7) | 0.74 | 6.6 ± 1.3 (4.1-9.2) | 0.96 |
|  | SAL | 13.3 ± 2.2 (8.8-17.7) |  | 74.7 ± 15.1 (44.2-105.3) |  | 6.5 ± 1.3 (3.8-9.2) |  |

*significant compared to SAL. CI: confidence intervals of the mean. s.e.m.: standard error of the mean. Note that injection hour -1 is the pre-injection hour. Hours 1-4 are the post-injection hours.
